# Improvement the Flame Retardancy and Thermal Conductivity of Epoxy Composites via Melamine Polyphosphate-Modified Carbon Nanotubes

**DOI:** 10.3390/polym14153091

**Published:** 2022-07-29

**Authors:** Xuejun Shi, Shiying Luo, Xiangxiang Du, Qingbin Li, Shiping Cheng

**Affiliations:** 1 School of Chemistry and Environmental Engineering, Pingdingshan University, Pingdingshan 467000, China; 2782@pdsu.edu.cn (X.S.); 2775@pdsu.edu.cn (S.L.); 2791@pdsu.edu.cn (X.D.); 2 Henan Key Laboratory of Germplasm Innovation and Utilization of Eco-Economic Woody Plant, Pingdingshan University, Pingdingshan 467000, China

**Keywords:** flame retardancy, epoxy composites, carbon nanotubes, surface chemical modification

## Abstract

Surface chemical modification of carbon nanotubes can enhance the compatibility with polymers and improve flame retardancy performances. In this work, the double bond active sites were constructed on the surface of carbon nanotubes modified by the γ-methacryloyloxypropyl trimethoxysilane (KH570). Glycidyl methacrylate (GMA) was further grafted onto the surface of carbon nanotubes via free radical polymerization. Finally, the flame retardant melamine polyphosphate (MPP) was bonded to the surface of carbon nanotubes by the ring-opening reaction. This modification process was proved to be achieved by infrared spectroscopy and thermogravimetric test. The carbon nanotubes modified by flame retardant were added into the epoxy matrix and cured to prepare flame retardant and thermal conductive composites. The flame retardancy of composites were studied by cone calorimetry, UL94 vertical combustion test and limiting oxygen index. The thermal conductivity of composites was characterized by laser thermal conductivity instrument. The results showed that when the addition amount of flame retardant MPP-modified carbon nanotubes in composites was 10 wt%, the flame retardant level of UL94 reached to V2, the limiting oxygen index increased from 25.1 of pure epoxy resin to 28.3, the PHRR of pure epoxy resin was reduced from 800 kW/m^2^ to 645 kW/m^2^ of composites and thermal conductivity of composites was enhanced from 0.21 W/m·K^−1^ of pure epoxy resin to 0.42 W/m·K^−1^ of the composites.

## 1. Introduction

Epoxy resin (EP) is one of an important thermosetting resin, which possesses many advantages, such as excellent electrical insulation, high mechanical properties, excellent solvent resistance and so on. It is widely used in the field of composites as electronics and appliances [1]. With the high integration of electronic devices, higher requirements are put forward for the flame retardant and thermal conductivity of epoxy composites. High flame retardant and thermal conductivity are an important development direction of epoxy composites [2,3].

The traditional thermal conductivity modification of epoxy resin is to realize the construction of thermal conductivity path via adding high thermal conductivity fillers, such as metal fillers, silver particles [4], silver nanowires [5] and copper particles [6]; or inorganic thermal conductivity particles, such as boron nitride [7], alumina [8], silicon carbide [9] and other powders; or carbon materials, such as graphene [10], carbon nanotubes and graphite; or the synergistic combination of them [11], and this method can enhance the thermal conductivity of epoxy composites. In order to obtain the flame retardant performance of epoxy composites, the modification way of intrinsic flame retardant could be adopted to make the epoxy resin molecules contain flame retardant elements, or the flame retardant curing agent was added in epoxy curing process. The flame retardant properties of epoxy composites were acquired through this method, and this kind of epoxy resin was inherently flame retardant, but its preparation process was complex, and it was not suitable for large-scale production, which limited the development and application of intrinsic flame retardant epoxy resin [12,13]. It was an effective strategy to solve the high cost of the flame retardant epoxy resin by directly additional flame retardant including DOPO [14], melamine salts [15] and other flame retardants, as well as nano powders [16], such as silica, montmorillonite [17], carbon nanotubes [18,19,20,21], graphene [3,22], carbon fiber [23] and so on. In particular, carbon nanotubes are one-dimensional quantum materials whose radial size is in the order of nanometers and axial size is in the order of microns. Both ends of the tubes are basically sealed. Compared with other nano materials, it has more unique structure and strange properties. It has high research value in many fields, such as high-efficiency solar energy converters, high-efficiency hydrogen storage materials, nano electronic devices, composite materials, conductive and thermal conductivity, nano flame retardants and so on [24,25,26].

These fillers endow the composites with the excellent flame retardant properties and mechanical and thermal conductivity properties. However, the addition of flame retardant to the composite will lead to the leakage and migration of flame retardant. The melamine salt flame retardant will exist in the epoxy resin in the mixed system in form of particles, which will reduce the mechanical properties of the composites [27,28,29]. In view of the high-performances requirements of thermal conductivity and flame retardancy of the epoxy resin, this work was based on carbon nanotubes, and it was modified by the silane coupling agent on its surface. Then, the glycidyl methacrylate (GMA) was grafted onto the surface of carbon nanotubes through polymerization, and the melamine polyphosphate (MPP) was bonded on the surface of carbon nanotubes via the ring-opening reaction to form flame retardant carbon nanotubes as the core, and the flame retardant MPP was the outer layer shell. The modified carbon nanotubes were added into the epoxy resin and cured by temperature programmed. Finally, the epoxy resin/carbon nanotube composites with the flame retardant and thermal conductivity were prepared. The purpose of this study was to strengthen the function of carbon nanotubes through surface modification in order to enhance flame retardancy and thermal conductivity of epoxy resin/carbon nanotube composites in the meantime. This functional modification strategy would provide a promising route to design epoxy composites with the enhanced flame retardant and thermal conductivity.

## 2. Experiment Part

### 2.1. Materials

Carbon nanotubes (CNTs, see Table 1), with the diameter of about 20 nm and a length of about 30~50 μm, were supplied by Henan national carbon nanotechnology Co., Ltd. (Pingdingshan, China).

The epoxy value of bisphenol A epoxy resin (EP, see Table 2) was 0.51, it was purchased from Shanghai McLean Biochemical Technology Co., Ltd. (Shanghai, China); 2-ethyl-4-methylimidazole (EMI-2,4, see Table 3), purity greater than 98%, was obtained by Shanghai McLean Biochemical Technology Co., Ltd.

The N-N dimethylformamide (DMF) was supplied by Sinopharm Chemical Reagent Co., Ltd. (Shanghai, China); azodiisobutyronitrile (AIBN) analytical reagent was purchased from Tianjin Fuchen Chemical Reagent Factory (Tianjin, China); glycidyl methacrylate (GMA), was purchased from Shanghai Titan Technology Co., Ltd. (Shanghai, China); absolute ethanol was supplied by Tianjin Yongda Chemical Reagent Co., Ltd. (Tianjin, China) Melamine polyphosphate (MPP) that was analytically pure was obtained by Shanghai McLean Biochemical Technology Co., Ltd. γ-methacryloyloxypropyl trimethoxysilane (KH570) was obtained by Shanghai Yuanye Biotechnology Co., Ltd. (Shanghai, China).

### 2.2. Preparation of Composites

#### 2.2.1. Carbon Nanotubes Modified by Silane Coupling Agent KH570

The carbon nanotubes (10 g) were added into a 1000 mL beaker and the dilute concentrated sulfuric acid and concentrated nitric acid were added into an acid solution with equal molar concentration, the diluted acid solution was mixed according to the volume ratio of 3:1, poured the mixed solution into the beaker and stirred magnetically for 5 h. The acidified carbon nanotubes were left to stand overnight; the supernatant was removed and washed by distilled water and then neutralized to neutral with sodium hydroxide solution, and the neutral solution was filtered and washed alternately with distilled water and ethanol three times. We put the acidified carbon nanotubes into a beaker, 2 mL KH570 was added into a certain amount ethanol, they were stirred fully and we put them into a vacuum drying oven at 80 °C for reaction for 4 h. After the reaction, the filter cake was repeatedly washed with ethanol and distilled water three times, and the filter cake was freeze-dried for 24 h to obtain KH570-modified carbon nanotubes CNTs-KH570.

#### 2.2.2. GMA Modified the CNTs-KH570

The modified CNTs-KH570 fillers (10 g) were added into a four port flasks equipped with a reflux condenser tube; 90 mL DMF and 10 mL GMA were added successively, and nitrogen was introduced for 30 min to discharge the air. Magnetic stirring in a 70 °C constant temperature water bath under nitrogen protection was done for 30 min, and the initiator AIBN 0.10 g (monomer concentration 10 wt%) was put in the flask. At the end of the reaction, the reactants were obtained by suction filtration, and then the filter cake was washed alternately with ethanol and acetone many times, and then the filter cake was freeze-dried to obtain the CNTs-KH570-PGMA particles with polymerized GMA on the surface of the CNTs.

#### 2.2.3. Flame Retardant MPP-Modified CNTs-KH570-PGMA

**Figure 1:**
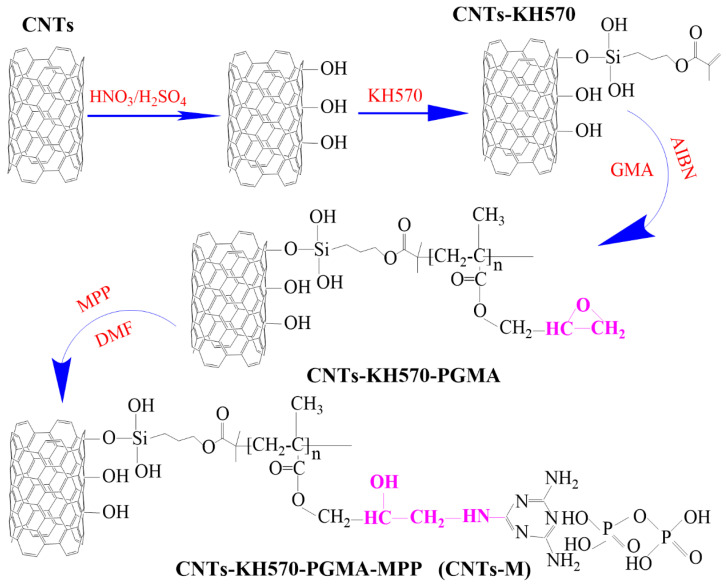
Schematic diagram of the preparation process of CNTs and their modified particles.

The CNTs-KH570-PGMA fillers (10 g) was added into the DMF solution containing 15 wt% MPP and soaked at room temperature for 24 h. The oil bath was heated and maintained 120 °C and reacted with magnetic stirring for 24 h. At the end of the reaction, the reactants were pumped and filtered to obtain filter cake, washed alternately with DMF and acetone three times and finally freeze-dried to obtain the MPP-modified CNTs-KH570-PGMA-MPP particles, abbreviated as CNTs-M. This preparation diagram is shown in Figure 1.

#### 2.2.4. Preparation of EP/CNTs-M Composites

A certain amount of CNTs-M was added into ethanol, and after ultrasonic dispersion and magnetic stirring for half an hour, the EP-51 was added into the solution. After ultrasonic stirring for half an hour, all the ethanol was removed by vacuum distillation under the heating condition of 50 °C. This solution replacement method can effectively avoid the agglomeration of carbon nanotubes in epoxy resin matrix [30]. After cooling to room temperature in an ice water bath, the curing agent EMI-2,4, measured according to 6 wt% of the mass of epoxy resin, was added, mixed and defoamed in a planetary mixer for 3 min; the operation was repeated three times to obtain an evenly mixed premix. Then the resin premix was poured into the stainless-steel mold with coating vacuum silicone grease and cured at 60 °C for 2 h and then 150 °C for 8 h. The pure epoxy resin and EP/CNTs-M composites were also prepared according to this process. The filling contents of CNTs-M in the composite EP/CNTs-M were 1 wt%, 3 wt%, 5 wt% and 10 wt% of the total mass of the composites, respectively, and the corresponding composites were abbreviated as EP/CNTs-M1, EP/CNTs-M3, EP/CNTs-M5 and EP/CNTs-M10, respectively.

### 2.3. Measurements and Characterization

The chemical structures of CNTs-KH570, CNTs-KH570-PGMA and CNTs-MPP were investigated by the tensor 37 Fourier transform infrared spectrum (Bruker Company, Ettlingen, Germany) using the KBr dilution pellets in the region of 400~4000 cm^−1^. The thermal analysis test was examined by the TA Q600 thermogravimetric analyzer (TA Instrument Company, New Castle, DE, USA). At the nitrogen atmosphere, the testing temperature range was from the 30~800 °C, and the heating rate was 10 °C/min. The sample morphology was observed by SU8010 field emission scanning electron microscope (Hitachi high tech company, Tokyo, Japan), and the scanning voltage was 1.0 kV. The powder sample was pasted on the sample table through conductive adhesive and was tested to observe its micro morphology. Based on GB/T 2408-2008 standard, UL94-X horizontal and vertical combustion tester (Modis China Combustion Technology Co., Ltd. Nanjing, China) was used to determine the combustion rate of the sample, and the sample size was 127 × 12.7 × 2.7 mm^3^. Based on GB/T 2406-2009 standard, HC-2C oxygen index tester of Nanjing Shangyuan Analytical Instrument Co., Ltd. (Nanjing, China), was utilized to confirm the limiting oxygen index (LOI) of the sample. The sample size was 100 × 10 × 4 mm^3^. Based on the GB/T 16172-2007 standard, the combustion performance of the sample was measured by using the cone calorimeter (FTT company, Derby, UK). The samples’ size was 100 × 100 × 2.7 mm^3^, and the radiant heat flux was 35 kW/m^2^. DLF-1200 laser thermal conductivity instrument (TA instrument company, New Castle, DE, USA) was employed to test the thermal conductivity of the samples. The samples’ size diameter was 25.4 mm, and the thickness was 2.30 mm. The samples were sprayed with graphite to treat the surface, and the test temperature was 30 °C.

## 3. Results and Discussion

### 3.1. Characterization of Flame Retardant Molecular Modification on the Surface of CNTs

#### 3.1.1. Infrared Spectrum

**Figure 2:**
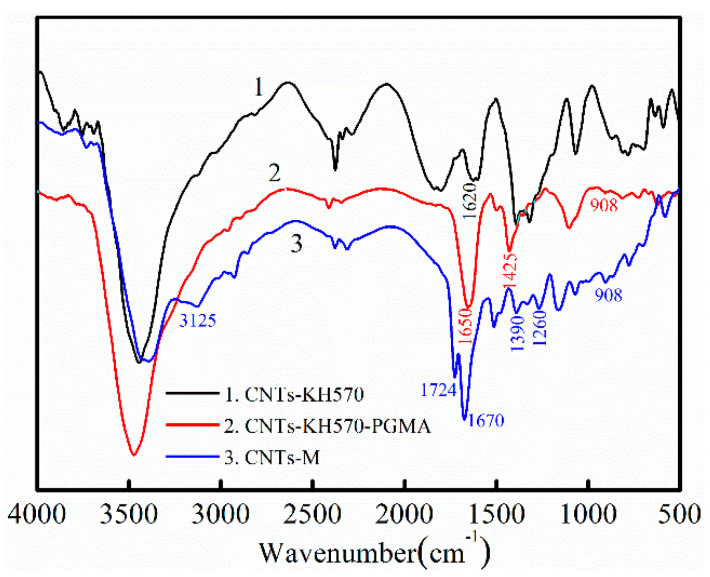
IR spectrum of (1) CNTs-KH570, (2) CNTs-KH570-PGMA and (3) CNTs-M.

The infrared spectra of the three particles are shown in Figure 2. Curve 1 represented the particles modified by silane coupling agent KH570, curve 2 displayed the particles CNT-KH570-PGMA polymerized on the surface of carbon nanotubes by GMA, and curve 3 displayed the infrared absorption curve of CNTs-M after the bonded flame retardant MPP. The absorption peak at 1620 cm^−1^ can be clearly seen from curve 1 in Figure 2, which was the absorption peak of C=C of carbon nanotubes modified by KH570. Combined with the TGA data of CNTs and CNTs-KH570, it can be seen that the surface modification method of carbon nanotubes was successful. As can be seen from curve 2 in Figure 2, the absorption peaks of the carbonyl group were at 1650 cm^−1^, the epoxy group was at 908 cm^−1^ and the methylene group was at 1425 cm^−1^; these results revealed that the GMA was grafted onto the surface of carbon nanotubes and formed CNTs-KH570-PGMA functional particles.

As can be seen from curve 3 in Figure 2, the shoulder peak of -NH_2_ appeared in 3125 cm^−1^, the vibration absorption peak of carbonyl group was at 1724 cm^−1^, the absorption peak of P-OH appeared at 1670 cm^−1^ and the absorption peak of -NH+ emerged at 1390 cm^−1^; the absorption peaks of the C-N key appeared at 1260 cm^−1^, which proved that melamine polyphosphate was bonded to the surface of carbon nanotubes. It was worth noting what emerged in the 908 cm^−1^ characteristic absorption peaks of the epoxy group, which showed that the grafting to the surface of carbon nanotubes PGMA were not all open loop. Probably because this part of the group package was buried by the PGMA polymer chains, these were not involved in MPP ring-opening addition reaction. Additionally, the epoxy groups were preserved in this part, which was helpful to enhance the binding force between carbon nanotubes and epoxy resin, and the thermal conductivity and mechanical properties of composites were improved.

#### 3.1.2. Thermogravimetric Analysis

**Figure 3:**
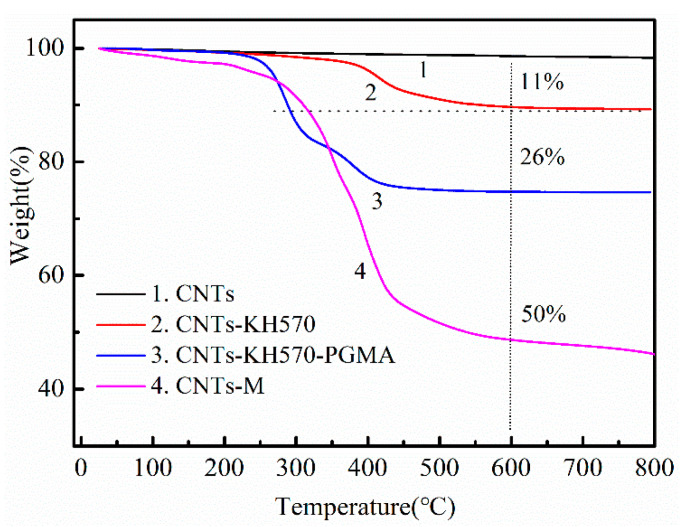
TGA curves of CNTs-KH570, CNTs-KH570-PGMA and CNTs-M.

Figure 3 shows the thermal weight loss curves of carbon nanotubes (curve 1), carbon nanotubes modified by a silane coupling agent KH570 (curve 2), carbon nanotubes polymerized by GMA (curve 3) and carbon nanotubes bonded by MPP (curve 4). Compared with curve 2, the grafting degree of silane coupling agent was about 11 g/100 g, compared with curve 3 and curve 2, the grafting degree of PGMA was about 15 g/100 g; compared with curve 4 and curve 3, the grafting degree of flame retardant MPP was about 24 g/100 g. The comparison of these results with the data of infrared spectroscopy showed that our design strategy was successful [31]. The MPP was bonded on the surface of carbon nanotubes via the ring-opening reaction, which established a foundation for improving the flame retardant performance of the composites in the next step.

#### 3.1.3. Morphology of CNTs and Modified CNTs

**Figure 4:**
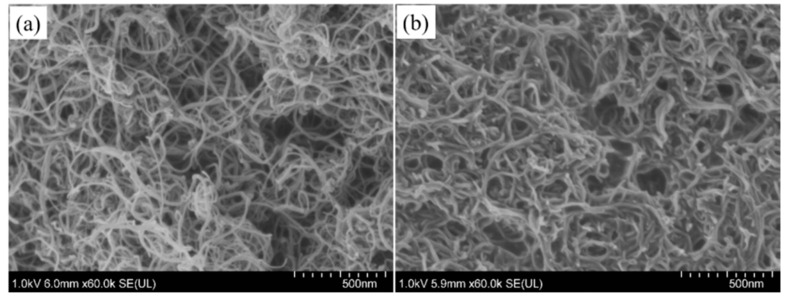
(**a**) SEM images of CNTs and (**b**) SEM images of CNTs-KH570-PGMA.

The sizes and morphologies of CNTs and CNTs-KH570-PGMA were observed by SEM in Figure 4. Figure 4a shows the SEM image of carbon nanotubes. It can be seen that the diameter of carbon nanotubes was about 30 nm and the length was about 30–50 μm in this work, and the surface boundary of single carbon nanotubes was relatively clear. In contrast, in Figure 4b, the polymer PGMA chains were grafted on the surface of carbon nanotubes, and the surface of carbon nanotubes became blurred under the high-energy electron bombardment of scanning electron microscope. Simultaneously, the diameter of the CNTs-KH570-PGMA had a slight growth; this phenomenon provided evidence that the surface of carbon nanotubes was modified by the PGMA polymer chains. According to the TGA data analysis in Figure 3 and the infrared spectrum data in Figure 2, the strategy of grafting polymer on the surface of carbon nanotubes was successful.

### 3.2. Thermal Stability of Composites

**Figure 5:**
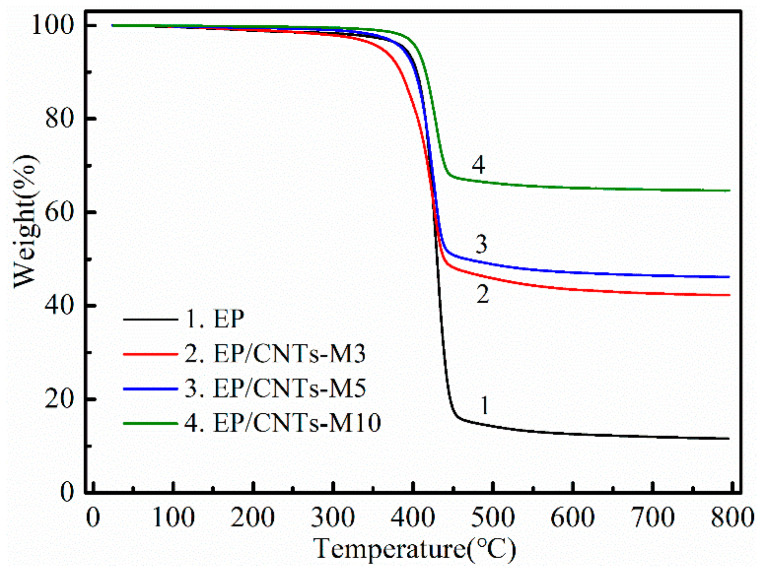
TGA curves of EP, EP/CNTs-M3, EP/CNTs-M5 and EP/CNTs-M10.

Figure 5 shows the thermogravimetric curves of epoxy resin and EP/CNT-M3, EP/CNT-M5 and EP/CNT-M10 composites under nitrogen atmosphere. In Figure 5, with the increasing of CNTs-M content, the residual amount of the composites becomes higher and higher. The residual amount of pure EP was about 10%, and when the amount of CNTs-M was 5 wt%, the residual amount of EP/CNTs-M5 composite was up to 50 wt%. When the addition amount of CNTs-M was 10 wt%, the residual amount of EP/CNTs-M5 composite reached 70 wt%, which further proved that the heat resistance of EP/CNTs-M composites were enhanced steadily with increasing of the addition amount of CNTs-M. These may be that the flame retardant MPP contents in the composites were gradually improved, and the reticular structure between carbon nanotubes could block the thermal decomposition of epoxy matrix. Meanwhile, the flame retardant MPP would also prevent the combustion of the composites and cause the decomposition of the composites matrix and then result in the improvement of the heat resistance of the composites.

### 3.3. Combustion Performance of Composites

The conical calorimeter is one of the effective methods for characterizing the combustion properties of materials under real fire conditions [32]. The flame retardant properties of composites were studied from three aspects in this work: energy change, smoke production and harmful gas.

#### 3.3.1. Study from the Heat Release

**Figure 6:**
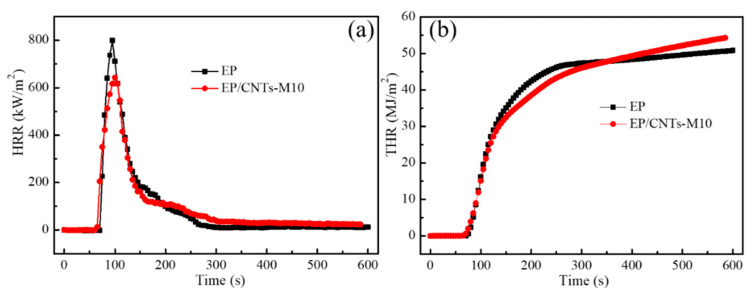
Heat release rate (**a**) and total heat release (**b**) versus time curves of EP and EP/CNTs-M10.

Figure 6 showed the heat release rate (HRR) and total heat release (THR) of epoxy resin and composites EP/CNTs-M10 with the combustion time. Figure 6a identifies that the pure EP was easy to burn, and its peak heat release rate (peak-HRR, PHRR) reached 800 kW/m^2^, while the PHRR of EP/CNT-M10 composites decreased to 645 kW/m^2^; it was 20% lower than that of pure EP. These results indicated that the CNTs grafted with flame retardant MPP can effectively reduce the heat release rate of the composites, and the flame retardant MPP was playing a flame retardant function, which can improve the flame retardant performance of the composites. It can be seen from Figure 6b that the total heat release of EP/CNTs-M10 composites was lower than that of pure EP during the 600 s of combustion. As the combustion process progresses, the flame retardants added to EP/CNTs-M10 composites were exhausted. In the later period of the combustion of the composites, the heat release of the composites was almost higher than that of the pure epoxy resin, which may mean that the polymer PGMA was grafted on the surface of the carbon nanotubes and was accompanied by a large amount of heat release, resulting in the total heat release of the composites EP/CNTs-M10 exceeding that of the pure epoxy resin. The results in Figure 6 could clearly prove that the carbon nanotubes modified by flame retardant MPP can indeed play a flame retardant function in the composites.

#### 3.3.2. Study in Terms of Smoke Production

**Figure 7:**
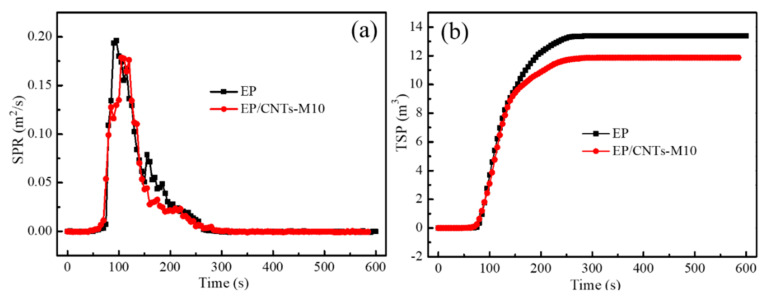
Smoke production rate (**a**) and total smoke production (**b**) versus time curves of EP and composites EP/CNTs-M10.

Figure 7 showed the smoke production rate (SPR) and total smoke production (TSP) of epoxy resin and composites EP/CNTs-M10 as a function of combustion time. As can be seen from Figure 7a, compared with pure epoxy resin, the smoke production rate of the composites EP/CNTs-M10 was decreased. The peak value of smoke production rate of pure epoxy resin appeared at about 100 s, while the peak value of smoke production rate of composite materials lagged behind to about 120 s, which proved that the smoke production rate of composites EP/CNTs-M10 modified with flame retardant can indeed be inhibited during the materials’ combustion. When the content of carbon nanotubes was 10 wt%, the peak smoke production rate of the composites EP/CNTs-M10 was decreased from 0.196 m^2^/s to 0.176 m^2^/s, and the value was decreased by about 11%. It proved that the carbon nanotubes grafted with flame retardant MPP can play a certain flame retardant function in the matrix of composites.

In Figure 7b, the total smoke production of EP/CNTs-M10 composite was lower than that of pure epoxy resin during the whole combustion process, and the total smoke production of EP/CNTs-M10 composites (11.7 m^3^) was always lower than that of pure epoxy resin (13.5 m^3^). This may be due to the function of carbon nanotubes and their surface-grafted flame retardants in EP/CNTs-M10 composites. In the process of burning materials, the flame retardant decomposed rapidly and produced a lot of not flammable (nitrogen, ammonia, vapor, etc.) to dilute the air near the combustible; polyphosphate also dehydrated to form more quickly in high temperature under the action of polyphosphoric acid, and it could promote dehydrated to form a carbide segregation layer to prevent the epoxy matrix composites from continuing to burn.

#### 3.3.3. Study from Harmful Gases

**Figure 8:**
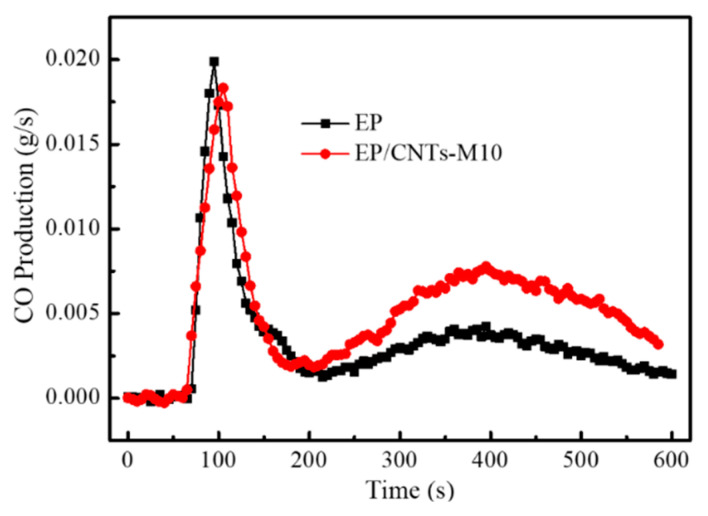
CO production rate versus time curves of EP and composites EP/CNTs-M10.

Figure 8 was the curve of CO generation rate over time during the combustion of epoxy resin and EP/CNT-M10 composites. It can be seen from the figure that the peak value of CO gas generated by composites EP/CNTs-M10 (110 s) lagged behind that of pure EP (100 s), which may be caused by the advance decomposition of the surface-grafted flame retardant MPP with carbon nanotubes to produce a large amount of non-combustible gas, which inhibited the combustion of the composite and causes it to not fully combust. However, the CO yield of the composites EP/CNTs-M10 was higher than that of the pure epoxy resin after 200 s, which may be attributed to the combustion of the polymer PGMA molecular chain grafted on the surface of carbon nanotubes. After the flame retardant burned for 200 s, a large number of non-combustible gases and carbonized layers covered the surface of the composites EP/CNTs-M10, resulting in low combustion efficiency and insufficient combustion of the composites. Therefore, after 200 s, the output of harmful gas CO of the composites EP/CNTs-M10 was higher than that of pure epoxy resin. This observation is often common for flame retarded polymer composites, which arises from decreased combustion efficiency leading to non-complete burning of the organic materials [33,34,35,36]. The peak value of CO gas produced by EP/CNTs-M10 composite was 0.018 g/s, which was lower than the peak value of CO gas produced by pure EP (0.02 g/s). These results also proved that the carbon nanotubes modified by MPP can reduce the production rate of harmful gas CO in epoxy composites.

### 3.4. Carbon Residue Diagram and SEM of EP and Composites

**Figure 9:**
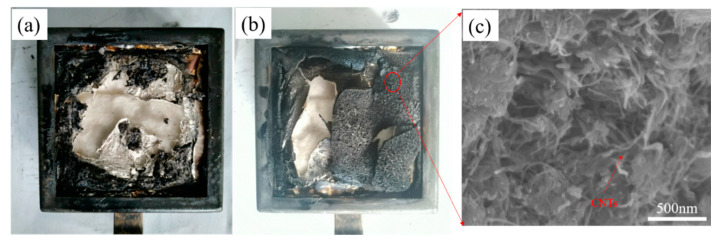
(**a**) Digital photos of carbon residue of EP and (**b**) composites EP/CNTs-M10; (**c**) SEM of carbon residue of composites EP/CNTs-M10.

Figure 9a,b shows digital images of the carbon residue of epoxy resin and composites EP/CNTs-M10. Figure 9c shows the SEM image of the carbon residue of composite material EP/CNTs-M10. It can be seen from Figure 9a,b that there was almost no residual carbon remained after conical calorimetric test combustion of pure epoxy resin. However, it can be clearly perceived from Figure 9b that a shell of residual carbon was formed after combustion of composites, and the main component was the composite of carbon nanotubes and residual carbon. The residual carbon of EP/CNTs-M10 composite was observed by SEM and the results is shown in Figure 9c. It can be found out that the most carbon nanotube morphology was maintained, and there were some residual blocks after the combustion of epoxy resin among the carbon nanotubes, which could prove that the carbon nanotubes modified by the flame retardants can play the function of the flame retardant filler. The flame retardancy of composites was enhanced.

### 3.5. Flame Retardant Performance Test of Composites

The results of limited oxygen index and UL-94 flame retardant grade of EP/CNTs-M composites are revealed in Table 4. The results showed that the limiting oxygen index of pure epoxy resin was 25.1, and the limiting oxygen index of the composites was enhanced with the increase of the addition amount of flame retardants modified carbon nanotubes. When the addition amount of CNTs was 10 wt%, the limiting oxygen index of the composite was up to 28.3. It was proved that the flammability of EP/CNTs-M composites was elevated. Meanwhile, the vertical combustion test results showed that the flame retardant grade of EP/CNTs-M10 composite reached V2, while the pure epoxy resin UL-94 flame retardant did not have a grade. This showed that the results of limiting oxygen index and UL-94 flame retardant grade test were consistent with the cone calorimeter results, and it also proved that the design strategy of carbon nanotubes surface-grafted flame retardant had certain feasibility.

### 3.6. Thermal Conductivity

**Figure 10:**
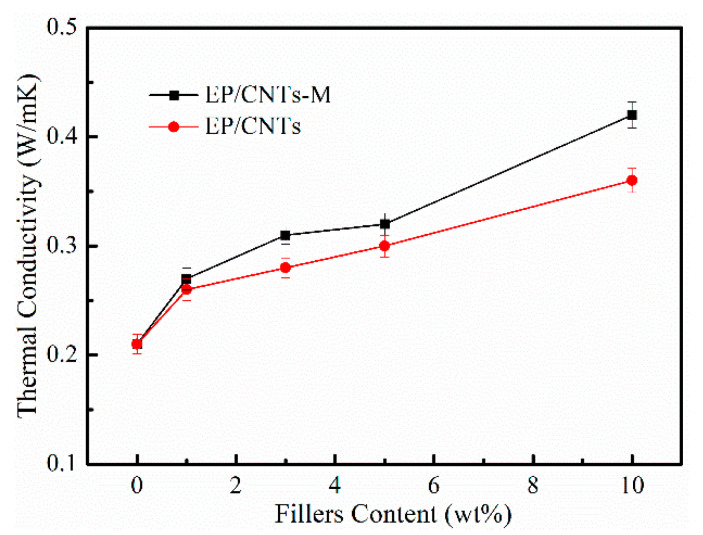
Thermal conductivity of EP and EP/CNTs-M with different content of CNTs-M.

Figure 10 showed the thermal conductivity of epoxy resin and composites EP/CNTs-M and EP/CNTs with different content of modified carbon nanotubes. In Figure 10, the thermal conductivity of pure EP was 0.21 W/m·K^−1^, while the thermal conductivity of the composites EP/CNTs-M and EP/CNTs were all enhanced. When the content of CNTs-M was 10 wt%, the thermal conductivity of the composites was increased to 0.42 W/m·K^−1^, which was two times higher than pure epoxy resin. As a control experiment, the thermal conductivity of composite EP/CNTs-10 was 0.36 W/m·K^−1^, and the thermal conductivity of composite EP/CNTs was always lower than that of composite EP/CNTs-M; this may be attributed to the fact that the surface modification of carbon nanotubes with MPP can improve the force between nanotubes and epoxy matrix, thus improving the thermal conductivity. However, the result did not reach to the ideal as expected, which may be due to the interface thermal resistance between carbon nanotubes and epoxy matrix, resulting in the thermal conductivity of the composites not being as good as we expected, which indicated that the surface modification of carbon nanotubes was indeed helpful to improve the thermal conductivity [37,38]. It also indicated that the thermal conductivity of composites could be enhanced via adding modified particles of carbon nanotubes into epoxy matrix, indicating that the surface modification of carbon nanotubes had a long way to go to reduce the interfacial thermal resistance between the carbon nanotubes and polymer matrix.

## 4. Conclusions

Molecular designed modification strategies were used in this work, and the EP/CNTs-M composites containing efficient flame retardant were prepared. The flame retardant properties of the composites were greatly improved in comparation with the pure epoxy resin. When the addition amount of flame retardant MPP-modified carbon nanotubes in the composites was 10 wt%, the flame retardant level of UL94 reached V2; the limiting oxygen index increased from 25.1 of pure epoxy resin to 28.3; the PHRR of pure epoxy resin was reduced from 800 kW/m^2^ to 645 kW/m^2^ of composites. Meanwhile, due to the reduction of the interfacial thermal resistance between the modified carbon nanotubes and EP matrix, the thermal conductivity of the EP/CNTs-M composites were also greatly enhanced from 0.21 W/m·K^−1^ of the pure epoxy resin to 0.42 W/m·K^−1^ of the composites. This functional modification strategy would provide a promising route to design epoxy composites with the enhanced flame retardant and thermal conductivity. These composites may be used in the field of electronic packaging with flame retardant and thermal conduction requirements, such as epoxy resin packaging adhesive for battery and its shell welding and other key regions.

## Figures and Tables

**Table 1:** Properties of CNTs.

The Qualitative Characteristics of CNTs	Value
Internal diameter (nm)	4~10
External diameter (nm)	10~30
Length (µm)	30~50

**Table 2:** Properties of epoxy resin E-51.

The Qualitative Characteristics of E-51	Value
Epoxy equivalent (g/mol)	192~216
Density at 25 °C (kg/m^3^)	1167
Molecular weight	375.86
Viscosity (Pa·s)	13~20

**Table 3:** Properties of EMI-2,4.

The Qualitative Characteristics of EMI-2,4	Value
Melting point (°C)	47~54
Density at 25 °C (kg/m^3^)	975
Molecular weight	110.16

**Table 4:** The limiting oxygen index (LOI) and UL-94 rating of EP and composites EP/CNTs-M.

Samples	EP	EP/CNTs-M5	EP/CNTs-M10
LOI (vol%)	25.1	26.4	28.3
UL-94 rating	NR	NR	V2

## Data Availability

The data presented in this study are available on request from the corresponding author.
